# Flow Profiles Identify Sources of Poor Metered Dose Inhaler Technique: An Observational Study in Patients with Long-Term Pressurized Metered Dose Inhaler Use

**DOI:** 10.1016/j.chpulm.2024.100116

**Published:** 2024-11-08

**Authors:** Stuart D. King, Rohan D. Milak, Hartmut Schneider, Mudiaga Sowho, Elizabeth C. Katz, Alan R. Schwartz

**Affiliations:** a Pulmonary and Critical Care Associates of Baltimore, MD, USA; b Gerresheimer Respimetrix GMBH Olten, Switzerland; c American Sleep Clinic Frankfurt, Germany; d Philipps University Marburg, Germany; e Division of Pulmonary Critical Care, Johns Hopkins School of Medicine, Baltimore, MD USA; f Towson University, Towson, MD USA; g University of Pennsylvania Perelman School of Medicine, PA USA; h Vanderbilt University School of Medicine, Nashville, TN USA; i Universidad Peruana Cayetano Heredia, San Martin de Porres, Peru

**Keywords:** asthma, bronchodilator drug delivery, COPD, inhaler technique, pMDI

## Abstract

**Background:**

Inhaled medications are commonly used as therapy for obstructive lung disease (OLD); however, poor technique from pressurized metered dose inhalers (pMDIs) can severely limit drug delivery and therapeutic efficacy. A novel flow sensor has been developed to characterize inhalation flow profiles from pMDIs.

**Research Question:**

Among patients with OLD, does recording flow profiles during pMDI inhalation maneuvers expose poor patterns of pMDI use, which can be used to remediate pMDI technique?

**Study Design and Methods:**

A novel flow sensor was coupled with a placebo pMDI to characterize inhalation technique in 70 participants with OLD from a pulmonary clinic. pMDI inhalation flow profiles generated actuation timing, mean inspiratory airflow, and inspired volume before and after visualizing these features. McNemar test was used to characterize the impact of training on pMDI inhalation metrics. The postactuation inspired volume was normalized to inspiratory capacity.

**Results:**

Among participants with a mean ± SD of 17.4 ± 17.9 years of pMDI use, flow profiles uncovered mistimed actuations in 47.1% and poor inspiratory flow rates in 30.0% of patients. After visualizing flow profiles, participants improved actuation timing (χ^2^ =12.042; *P* < .001), mean inhaled volume (87.9% to 105.6% of inspiratory capacity; *P* < .001), and the combined inhalation metrics (χ^2^ =8.45; *P* = .003**).**

**Interpretation:**

Flow profiles helped uncover and remediate specific defects in pMDI technique in most chronic users with OLD. Flow profiles from inhaled medications can be used to enhance drug delivery to the lung.

**Clinical Trial Registration:**

ClinicalTrials.gov; No.: NCT05495256; URL: www.clinicaltrials.gov

**Take-Home Points**

**Study Question:** Can flow profiles be used in patient with obstructive lung disease to identify and correct poor pressurized metered dose inhaler (pMDI) technique?

**Results:** Our results showed that using flow profiles helped patients identify and correct poor pMDI technique, specifically actuation timing and adequate inhaled volume.

**Interpretation:** This study showed that a large proportion of patients with obstructive lung disease have poor pMDI technique that can be improved by using inhalation flow profiles to provide biofeedback.

Obstructive lung disease (OLD) consists of a spectrum of disorders including asthma and COPD, resulting in acute exacerbations and debilitating chronic respiratory symptoms. Airflow obstruction contributes significantly to excess morbidity and mortality in both disorders,^1^, ^2^, ^3^ loss of productivity, and excess health care expenditures.^1^^,^^4^, ^5^, ^6^ Inhaled medications remain a common therapy modality across the entire range of asthma and COPD severity, and reduce respiratory symptoms, improve lung function, and can prevent and treat exacerbations. The medical and financial burden of inhaled bronchodilators is substantial but can only be justified if adequate drug deposition in the lung can be assured.

The pharmacologic benefit of inhaled medications is not harnessed by a large proportion of patients who are not using their prescribed inhalers correctly.^7^, ^8^, ^9^ Several steps have been taken to optimize pressurized metered dose inhaler (pMDI) use. First, guidelines and training materials have been developed to train patients in performing a sequence of steps required to optimize delivery of inhaled medications.^10^, ^11^, ^12^, ^13^ However, more than one-half of patients continued to use their inhaler improperly. Second, monitors have been developed with the goal of improving adherence to bronchodilator medications, with built-in counters on dispensers and patient diaries to record and track the number and time of actuations.^14^, ^15^, ^16^, ^17^ Third, digital platforms have facilitated data transmission and log when patients actuate their devices, but do not assess technique.^18^, ^19^, ^20^ Fourth, methods have been promulgated to track the duration and pace of the inhalation maneuver, which provide indirect measures of drug administration.^21^, ^22^, ^23^, ^24^ Examples of devices that measure inspiratory flow rate include spirometry and the In-Check Dial (Alliance Tech Medical, Inc).^25^, ^26^, ^27^ Thus, despite efforts to standardize guidelines, monitor adherence, and assess technique, a substantial proportion of patients continue to use pMDIs incorrectly.

To date, no single technology can characterize a patient’s use, actuation timing, and inhalation depth and speed. We developed a novel flow sensor, which when coupled with a pMDI can profile a patient’s pMDI actuation and inspiratory airflow. Current guidelines suggest that mean flow rates in the range of 30 to 90 L/min are required to ensure the medication is delivered to the lung rather than deposited in the upper airways.^9^^,^^11^^,^^14^^,^^26^^,^^28^ Patients must also time their actuation at the start of inhalation to ensure adequate penetration of aerosolized medication into the lungs.^13^^,^^28^^,^^29^ Proper technique also requires the patient to inhale fully to maximize medication delivery to the lower airways.^14^^,^^28^^,^^29^ Finally, patients must hold their breath for up to 10 seconds after inhalation to allow time for gravitational sedimentation of medication.^30^^,^^31^

The aforementioned considerations led us to suspect that among participants with a range of OLD severity and years of use, flow profiles would reveal a spectrum of patterns of poor pMDI use. We further hypothesized that once specific errors in pMDI actuation timing and/or inhalation flow and volume were identified, patients could recognize these deficits and change their inhalation technique accordingly. To address this aim, an observational study was conducted in which flow profiles were assessed during pMDI actuation of a placebo inhaler in participants with asthma and COPD actuation timing, and inspiratory flow and volume were characterized during use and after providing feedback on usage patterns.

## Study Design and Methods

### Participant Selection

A convenience sample of new and established patients with a history of OLD was recruited from 2 outpatient pulmonary offices in the Baltimore, Maryland, region. Patients with a diagnosis of asthma and/or COPD who used at least 1 pMDI were eligible as participants for this study. Patients in whom inhaler use was contraindicated or who had a tracheostomy were excluded. This protocol was approved by the Advarra institutional review board, and informed consent was obtained from all participants before enrollment in the protocol (ClinicalTrials.gov registration No. NCT05495256).

### Experimental Setup

**Figure 1:**
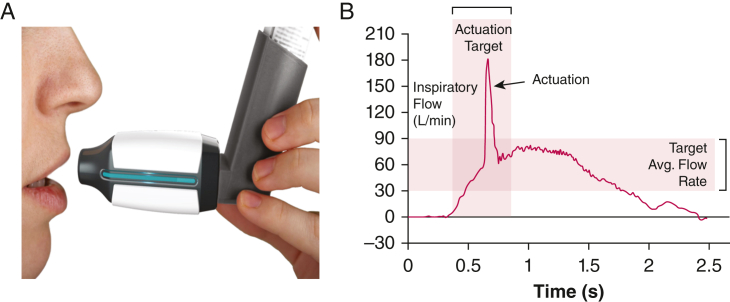
A, B, iQhaler apparatus for generating a representative inspiratory flow profile. A, Picture of the iQhaler device coupled with a pressurized metered dose inhaler filled with placebo medication. B, Illustrative example of the flow profile generated from the device. This graph depicts inspiratory flow vs time. The horizontal shaded region represents the target mean flow rate between 30 and 90 L/min. The vertical shaded region shows the ideal time of actuation in the first third of the inhalation. Avg = average.

A novel flow monitor device (iQhaler; Gerresheimer Respimetrix GmbH) was coupled in series to a pMDI filled with placebo medication (Fig 1A). The device measures 8.255 cm long, 3.81 cm wide, and 3.81 cm in height. The sensor incorporated a previously validated flow monitoring mechanism, based on the Pitot principle that measures pressure in the center of a tube computing pressure differences between a proximal and a distal reference port.^32^^,^^33^ A MEMS pressure sensor (Model SDP31-500PA-TR-250PCS; Sensirion AG) was used to calculate the airflow over the course of each inhalation maneuver with a high level of accuracy of 0.03% with the flow range from −180 and 180 liters per minute. The device was configured to record flow within an extended range of ± 207.5 liters per minute.

The iQhaler was configured to start acquiring the flow signal at a sampling frequency of 100 Hz whenever the participant’s lips made and maintained contact with the mouthpiece which was visually confirmed by the research staff. The device recorded the patient’s airflow signal for up to 8 seconds, thereby capturing the entire inhalation flow curve. It then stored the data and transmitted the deidentified flow profile via Bluetooth into a database. The database was accessed via web portal to display and analyze flow profiles from each attempt (Fig 1B).

### Study Protocol

Patients were asked to perform 2 series of 2 inhalation maneuvers. In the first series of inhalations, the patient was asked to demonstrate his or her usual inhalation technique through the iQhaler 3 times evenly spaced by several minutes, while blinding the participant to the flow profile generated by the device. Patients then performed a minimum of 3 slow vital capacity maneuvers with a handheld spirometer (ndd Medical EasyOne Air Spirometer; ndd Medical Technologies), which provided measurements of vital capacity and inspiratory capacity (IC). IC is the maximum volume that can be inhaled after reaching the end of a normal exhalation.^34^

Immediately thereafter, patients were allowed to visualize their pretraining iQhaler flow curves and provided with feedback based on objective metrics (actuation timing, inhalation flow rate and the volume during the inhalation maneuvers) (Fig 1B). These metrics were used to describe the adequacy of the patient’s technique. Patients were then shown an instructional video (https://www.youtube.com/watch?v=fHYTz-ZoRLw), which demonstrated standard proper 8-step inhaler technique how to use metered dose inhaler.^35^

Patients then practiced their technique with the placebo pMDI connected to the iQhaler. The median time of training was 24 minutes and 6 seconds (interquartile range, 14 minutes 35 seconds to 33 minutes 53 seconds). The impact of visualizing inhalation flow curves during the pMDI training exercise and pMDI dispensing technique was then assessed by having participants repeat 2 inhalation maneuvers through the placebo pMDI and iQhaler device.

### Characterization of the Flow Profile

The iQhaler recorded a flow curve over time and calculated the inspired volume over the duration of the inhalation maneuver. The actuation could be readily identified in the patient’s flow curve and was represented by a brief spike in flow during the maneuver (Fig 1B**)**.

The following criteria were used to characterize the adequacy of the participants’ technique.

#### Actuation Timing

The timing of actuation was assessed with respect to the duration of the inhalation. Actuations that occurred in the first third of the inhalation were defined as good in alignment with Global Initiative for Chronic Obstructive Lung Disease standards, as shown in Figure 1B.^36^^,^^37^ Actuations occurring before or after the first third of inhalation were classified as poor. Failure to actuate the pMDI or multiple actuations during a single inhalation were also considered poor. Profiles in which the actuation was undetectable were labeled as poor. Undetectable actuations occurred when the patient did not manually actuate the inhaler, when their lips were not in contact with the inhaler, or when the actuation spike was subsumed by excessive inspiratory flow.

#### Mean Inspiratory Flow

As illustrated in Figure 1B, the peak inspiratory flow spikes during the actuation of the placebo pMDI. As such, the device in its present form does not distinguish between the peak inspiratory flow generated by the patient and the actuation spike. We performed a subanalysis of our data set and determined that peak inspiratory flow rate and mean inspiratory flow (MIF) were significantly correlated (Pearson = 0.607; *P* < .001). MIF between 30 and 90 L/min was deemed good, consistent with guidelines for pMDI flow rates.^28^^,^^38^, ^39^, ^40^ Flows below or above this range were considered poor.

#### Inhaled Volume

The volume of inhalation through the iQhaler was calculated as area under the curve. To account for potential errors of the volume added by the actuation, a subset analysis was performed to assure that the area under the curve reflects the inhaled volume. The actuation volume was subtracted from the overall volume of the flow profile. Using a paired *t* test, we compared the volumes in 20 randomly selected recordings with vs without the spike and found no significant difference (2.90 vs 2.81; *P* = .23). Inhaled volume was assessed relative to the IC, as measured by spirometry. Volumes that were > 90% of the IC were considered optimal, whereas volumes below this level were considered poor.

Inhalation maneuvers were defined as good technique if patients met all 3 criteria as previously defined. Poor technique was defined by inadequate performance with respect to any of the metrics (ie, flow, timing, volume).

### Data Analysis

To address the main hypothesis that flow profiles reveal specific errors in bronchodilator technique (viz, actuation timing, inhalation flow, volume), descriptive statistics were performed to examine the frequency of poor pMDI use before the training exercise. Specifically, univariable statistics were used to describe the actuation timing (relative to the inhalation duration), MIF, and inhalation volume before and after training.

To determine whether training improved the volume pretraining and posttraining, a Student paired *t* test was used to compare mean inhalation volumes and a Pitman-Morgan paired *t* test compared the variance in inhalation volumes. In these comparisons, inhalation dose volume was normalized to the patients’ IC. For categorical outcomes, McNemar test was also used to detect a training effect in paired pretraining and posttraining samples.

A goodness of fit χ^2^ test was conducted to test whether the observed proportions of participants in each group differed from the expected proportion (ie, that there would be equal numbers of participants in all 3 groups). All analyses were performed in SPSS. Statistical inferences were made when *P* < .05.

## Results

### Participant Characteristics

Participants’ demographic and anthropomorphic characteristics appear in Table 1. The cohort consisted of predominantly older women with moderate obesity and a diagnosis of moderately severe asthma, COPD, or overlap syndrome. Patients had a longstanding history of OLD spanning 1 to 3 decades. They reported a history of inhaled bronchodilator therapy for a similar duration. Less than one-half recalled having been trained to use inhaled bronchodilators, and the remainder reported training experiences that were limited in duration. No difference in baseline technique was detected between participants who reported prior (n = 30) training compared with those who did not (n = 17; *P* = .133).

**Table 1:** Patient Characteristics

Parameter	Value			
	Total (N = 70)	Asthma (n = 36)	COPD (n = 25)	COPD + Asthma (n = 9)
% Female	79.5	84.6	72.0	77.8
Age, y	64.7 [14.2]	60.4 [16.7]	71.2 [6.8]^a^	65.9 [12.4]
BMI, kg/m^2^	31.4 [7.9]	33.0 [8.2]	29.5 [7.4]	29.3 [6.7]
Disease severity				
FEV_1_, L	1.8 [0.7]	1.9 [0.8]	1.5 [0.7]	1.8 [0.6]
FEV_1_ predicted	76.0 [25.0]	85.2 [21.5]	66.8 [23.4]	79.2 [19.5]
FVC, L	2.7 [0.9]	2.7 [0.9]	2.6 [1.0]	3.0 [0.8]
FVC predicted	85.1 [20.0]	85.2 [21.5]	82.9 [19.3]	90.0 [16.7]
FEV_1_/FVC	0.64 [0.1]	0.69 [0.1]	0.57 [0.1]	0.64 [0.1]
Baseline IC, L	2.2 [0.8]	2.1 [0.6]	2.1 [0.9]	2.6 [1.0]
Baseline SVC, L	2.8 [0.9]	2.7 [1.0]	2.8 [0.8]	3.2 [1.1]
Duration of disease, y	17.6 [18.5]	24.2 [20.5]^a^	8.7 [12.6]	13.3 [11.2]
Medication use				
SABA/SAMA, %	100.0	100.0	100.0	100.0
ICS, %	68.5	71.8	60.0	77.8
LABA, %	65.8	59.0	72.0	77.8
LAMA, %	41.1	15.4	76	55.6
Average No. of inhaler medications	2.8 [1.0]	2.5 [0.9]	3.1 [1.2]	3.1 [0.8]
Oral steroids, %	1.4	2.6	0.0	0.0
Biologics, %	6.8	10.3	0.0	11.1
Azithromycin (prophylactic), %	1.4	0.0	4.0	0.0
Roflumilast, %	0.0	0.0	0.0	0.0
Montelukast, %	27.4	38.5	12.0	22.2
Average No. of noninhaled pulmonary medications	0.3 [0.6]	0.5 [0.7]	0.1 [0.3]	0.3 [0.7]
Inhaler history				
Duration of inhaler use, y	17.4 [17.9]	23.6 [19.9]	9.5 [12.6]	13.2 [10.9]
Received training, %	41.0	46.2	28.0	55.6
Reported amount of inhaler training, min	5.3 [10.2]	3.8 [4.7]	6.6 [16.3]	8.6 [11]

Values are mean [SD] or as otherwise indicated. IC = inspiratory capacity; ICS = inhaled corticosteroid; LABA = long-acting beta-agonist; LAMA = long-acting muscarinic antagonist; SABA = short-acting beta-agonist; SAMA = short-acting muscarinic antagonist; SVC = slow vital capacity.

a Significant differences between groups were seen in age (COPD greater than asthma, *P* = .010), duration of diagnosis (asthma greater than COPD, *P* = .002), and prevalence of LAMA usage (COPD greater than asthma, *P* < .001).

#### Pretraining Timing Patterns and Flow Rates

**Figure 2:**
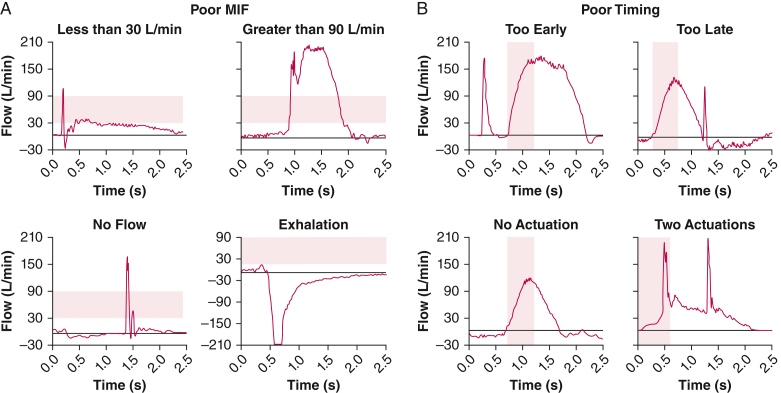
Illustrative patterns of misuse. The horizontal shaded region in the top row represents the target mean flow rate (30 to 90 L/min). The vertical shaded region in the bottom row represents the ideal timing of actuation (first third of inhalation). A, examples of MIF outside the target range (shaded horizontal region). B, examples of timing outside of target range (shaded vertical region). Actuation of pressurized metered dose inhaler can be seen as a sharp spike in the flow profile. MIF = mean inspiratory flow.

In Figure 2B, graphs of inhalation flow vs time illustrate several patterns of poor actuation timing that were observed pretraining. In each example, the timing of the actuation spike fell outside the actuation target range (shaded region on each flow profile) or did not appear in the flow profile whatsoever when the patient failed to actuate the pMDI. Several distinct patterns of actuation timing were discerned in which the actuation was either too early or too late (upper panels). Alternatively, some maneuvers demonstrated either no actuation or multiple actuations during the inhalation (lower panels). Also observed were partial or absent flow profiles when lips did not contact the mouthpiece (subsequently discussed).

In Figure 2A, several patterns of poor inhalation flow rates are illustrated before training. Each graph depicts the inhalation flow vs time for a single maneuver. Relative to the target flow rate range (shaded region of each flow profile), the peak inspiratory flow was either too low or too high (upper panels). In other cases, the inspiratory flow rate was either absent or the patient mistakenly exhaled rather than inhaled (lower panels).

#### Flow Profiles Before Training and After Training

In Table 2, the proportion of participants demonstrating specific poor timing and mean flow patterns appears for pretraining and posttraining maneuvers. A relatively large percentage of participants demonstrated poor inhalation technique before training. These patterns revealed a high proportion of mistimed actuations in nearly one-half of patients and mean inspiratory flow rates outside the target range in over one-fourth of patients. Training resulted in reductions in the proportion of patients within most categories of timing and poor mean flow except for those with early actuations and excess inspiratory flow, respectively.

**Table 2:** Technique Before Training vs After Training (N = 70)

Mean Inspiratory Flow			
Good use	Pre (%)	Post (%)	*P* Value
Between 30 and 90 L/min	**68.6**	**79.3**	.112
**Poor use**			
< 30 L/min	7.1	6.4	
> 90 L/min	18.6	12.6	
Nasal breath	1.4	0.0	
Exhalation	2.9	0.0	
No contact	1.4	1.4	
Combined poor use	**31.4**	**20.7**	**.112**

**Actuation Timing**			

Good timing			
First third	**52.1**	**74.3**	< .001
**Poor timing**			
Early	12.1	13.6	
Late	14.3	5.7	
No detectable actuation	17.1	5.0	
Multiple actuations	2.1	0.0	
No contact	2.1	1.4	
Combined poor timing	**47.9**	**25.7**	**< .001**

**Volume**			

**Good volume**			
> 90% IC	37.7	71.0	**< .001**
**Poor volume**			
< 90% IC	62.3	29.0	**< .001**

IC = inspiratory capacity. Bold font indicates subcategories related to poor use/timing/volume.

**Figure 3:**
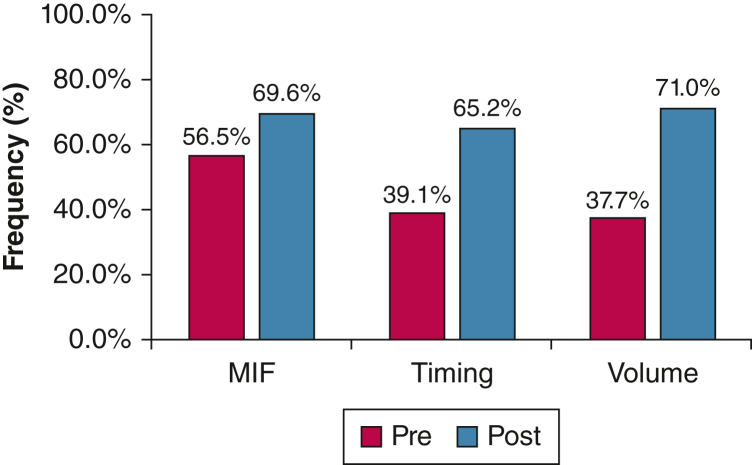
Impact of training. Percentage of participants with acceptable MIF, timing, and inhaled volume > 90% of inspiratory capacity pretraining (red) and posttraining (blue). Compared with pretraining inhalations (red bars), accuracy in hitting the target MIF range (*P* > .05) did not change, whereas actuation timing (c^2^_1_ = 12.042; *P* < .001) and volume (c^2^_1_ = 17.926; *P* < .001) improved posttraining. MIF = mean inspiratory flow.

In Figure 3, the impact of training on the frequency of correctly performed timing and mean flow is illustrated. Compared with pretraining inhalations (red bars), MIF actuation timing (χ*2* = 12.042; *P* = .02) and actuation timing (χ^2^ = 17.926; *P* < .001) improved posttraining.

**Figure 4:**
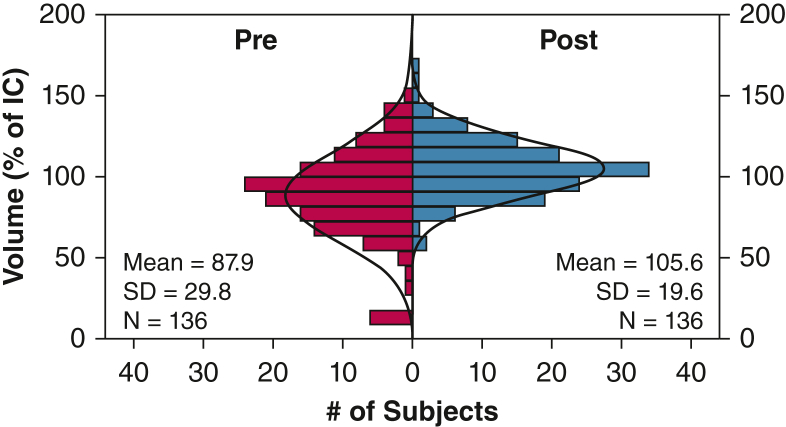
Distribution of iQhaler inhaled volume normalized to IC before training and after training. Training increased mean volume (87.9% vs 105.6%; *P* < .001) and reduced SD (29.8 vs 19.6; *P* < .001). IC = inspiratory capacity.

In Figure 4, the inhaled volume during pMDI inhalation maneuvers is represented as a percent of the patients’ IC before and after the training exercise. Training was associated with an increase in the mean inhaled volume from 87.9% to 105.6% of IC (*P* < .001) and a decrease in the SD from 29.8% to 19.6% (*P* < .001).

When categorized into 3 groups (worsened, no change, or improved), we found that the expected and observed values differed significantly (χ^2^ = 21.48; *P* < .001). These results demonstrated that more participants than expected either improved or stayed the same after training and fewer participants than expected worsened.

**Figure 5:**
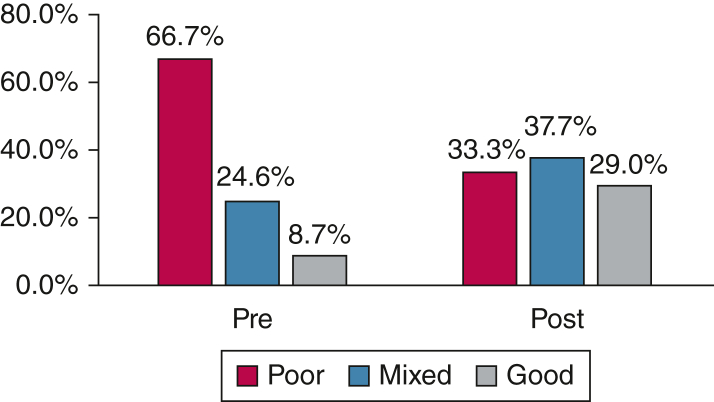
Frequency of participants performing all 3 paramaters correctly (mean inspiratory flow, timing, and volume) before training (red) and after training (gray). Participants that met all 3 metrics (mean inspiratory flow, timing, and volume) on both pretraining or posttraining attempts were categorized as good, on one attempt were categorized as mixed, and on both attempts were categorized as poor. Training significantly increased the frequency of participants who demonstrated good use from pretraining to posttraining (c^2^_1_ = 8.45; *P* = .003).

In Figure 5, patients’ performance is described before training and after training. Before training, most patients performed poorly with a substantially lower proportion of participants partially or completely failing to meet the performance goal. After training, a considerable improvement in performance was observed with most patients achieving partial (mixed) or good performance.

Of note, training significantly increased the frequency of patients who demonstrated good use on all 3 quality metrics (mean inspiratory flow, timing, and volume) from before training to after training (χ2 = 8.45, *P* = .003) (Fig 5).

## Discussion

In this study, a novel flow sensor was used to characterize flow profiles while patients with chronic use pMDI use inhaled and actuated a placebo device. Flow profiles revealed a preponderance of poor pMDI maneuvers in an experienced cohort with defects in inhalation flow rate and actuation timing, leading to substantial reductions in postactuation inhaled volume. Several major findings are reported in this study. First, poor flow rates were observed, with many participants inhaling too fast, whereas others demonstrated a complete lack of drug delivery by inhaling through their nose or exhaling instead of inhaling. Second, flow profiles revealed significant difficulty coordinating the actuation timing with inhalation. Participants actuated either too early or too late, and in several instances failed to actuate or actuated multiple times. Third, flow profiles provided potentially valuable feedback that could be used to improve their inhalation technique. Our findings imply that poor pMDI technique is commonplace among long-term users, and that monitoring flow profiles with feedback may add value to existing pMDI training efforts.

Several devices already couple with pMDIs for the purpose of monitoring adherence and technique. These devices incorporate counters and time stamps to track pMDI usage, and have generally revealed unusual pMDI usage patterns.^10^^,^^21^^,^^25^^,^^41^ More recently, devices have incorporated sensors to report the duration of inhalation and timing of actuation to optimize drug delivery.^21^^,^^25^^,^^41^ These features are further extended by the present device, which generates the entire flow profile during the inhalation maneuver. Characterizing these flow profiles offered several novel insights. First, flow profiles revealed poor inhalation flow rates and actuation timing in a large proportion of patients. Second, poor timing in the patients reduced their inspired volume, such that drug delivery to the lungs would be severely reduced in most patients. Third, many of the patients, who had been using inhalers for decades, were not immune from poor use. In fact, we were surprised to find that flow profiles in some patients displayed defects in technique that resulted in little to no delivery of medication to the lungs, highlighting an egregious waste of medication in a large proportion of patients.

Our findings also highlight a critical unmet need for training patients to optimize flow rate, actuation timing, and volume metrics when they use a pMDI (Table 2). Flow profiles allowed us to target and remediate specific defects in pMDI administration that could ultimately increase drug delivery, increase inhaled therapy efficacy, and improve disease management over time. When patients visualized their flow profiles in this study, they could identify and correct specific flaws in their inhalation technique. Incorporating graphic feedback from patients’ flow profiles helped them optimize their pMDI technique. These flow profiles also revealed persistent defects in inhalation technique in a substantial proportion of patients who could not develop the skills necessary to ensure adequate, consistent pMDI drug delivery. Identifying persistent defects in flow profiles can prompt health care providers to consider other options for ensuring medication delivery (eg, valve holding chambers, dry powder inhalers, nebulized treatments).

The iQhaler extends capabilities of predicate devices used for assessing pMDI inhalation technique. Rather than generating quantitative flow profiles, the Trainhaler (Clement Clarke) provides semiquantitative audio feedback to help patients maintain proper flow rates during pMDI inhalation. Trainhaler monitoring demonstrated that most patients inhaled too fast during the maneuver, and could incorporate this feedback to improve technique.^42^ Using the flow profile, we extended the approach by detecting several types of poor technique and tracking inhaled volume with the iQhaler. The AIM Vitalograph device also measures inspiratory flow rate during the pMDI maneuver, actuation timing, and breath-hold time at end of inhalation. Studies with the AIM device in patients with asthma have also shown poor technique in 58.6% of those tested, similar to the results with the iQhaler.^43^ This device however is configured for stationary assessments at point of care rather than portable monitoring of inhalation technique in daily routine (as would be possible with the iQhaler).

The major limitation of our study is that it was observational by design with its primary goal of characterizing flow profiles to provide insight into participants’ inhalation technique. Several other limitations should also be considered in interpreting our findings. First, we acknowledge the lack of a parallel control group in our intervention trial; however, the design is strengthened by the fact that each patient served as his or her own control. Second, our study was conducted in a relatively small convenience sample of patients with OLD. Although the sample included patients with a broad range of COPD and asthma, our findings may be limited to relatively stable outpatients on pMDI treatment. Third, our study only examined responses to a short-term training exercise, whereas the long-term impact of the intervention remains unknown. Fourth, we did not distinguish between the impact of a training video demonstrating technique vs visual feedback from patients’ own flow curves. Nonetheless, the proportion of patients with poor use at baseline did not differ between those with and without previous pMDI training, suggesting the need for ongoing reinforcement of usage technique. Fifth, we recognized that an actuation spike in the flow profile was not apparent in a limited number of curves, thereby limiting our ability to characterize the actuation timing. Nevertheless, the training exercise improved our ability to detect the actuation spike (from 17.1% to 5.0%) (Table 2) and determine the actuation timing, suggesting that the actuation spike may be a marker of effective pMDI use. Finally, instructions such as shaking and holding upright before/during inhalation and a 10-second breath hold after the maneuver were not captured with the device.

Flow profiles generated by a simple, lightweight novel flow sensor coupled with a pMDI indicated that a large proportion of patients with OLD do not use prescribed pMDI medications correctly. Visualizing the flow profile may add value in training patients in effective pMDI technique and provide them with the ability to monitor their technique over time. We found a remarkably high prevalence of poor pMDI that is even higher than previously reported and likely limits lung deposition of bronchodilators.^44^, ^45^, ^46^, ^47^, ^48^ Reductions in drug delivery could drive increased rates of exacerbations and hospitalizations, and accelerate decline in pulmonary function.^21^^,^^37^^,^^38^ The flow profile could provide a vital tool for focusing the patient on correcting specific defects in pMDI use, which can enhance drug delivery and help maintain lung function and quality of life over time. Work still needs to further characterize inhalation patterns by adding additional features to determine whether patients shake the device, correctly orient the pMDI, maintain a 10-second breath hold after actuation and to compare the iQhaler with other pMDI monitoring devices. Further studies in which flow profiles are compared with established guidelines^36^^,^^49^^,^^50^ for pMDI technique will ultimately be required to determine whether these add value to clinical practice and can be used in daily routine without altering the aerosol composition.

## Funding/Support

This study was supported by Gerresheimer Respimetrix GmbH.

## Financial/Nonfinancial Disclosures

None declared.

## Supplementary Material

e-Online Data
