# Supplementary material for: Flow Profiles Identify Sources of Poor Metered Dose Inhaler Technique: An Observational Study in Patients with Long-Term Pressurized Metered Dose Inhaler Use
Source: CHEST Pulm. 2024 Nov 8;3(3):100116. doi: 10.1016/j.chpulm.2024.100116 (PMC13417802; doi:10.1016/j.chpulm.2024.100116)
Supplement: e-Online Data [file mmc1.docx]

e-Table 1

| **e-Table 1** | | | |
| --- | --- | --- | --- |
| **Mean (SD) of Inhalation Metrics Pre- Vs Post-Training** | | | |
|  | **Pre** | **Post** | **P Value** |
| Duration (s) | 1.67 (0.76) | 2.05 (0.71) | p < 0.001 |
| PIF (L/min) | 116.7 (41.0) | 123.5 (34.3) | p = 0.132 |
| Volume (% of IC) | 87.9 (29.8) | 105.6 (19.6) | p < 0.001 |

Significant changes in duration (1.67 vs 2.05 seconds, p < 0.001) and

Volume (87.9 % vs 105.6% of IC, p < 0.001) were found. No difference

in PIF was seen (116.7 vs 123.5 L/Min, p = 0.132).
